# The furosemide stress test: current use and future potential

**DOI:** 10.1080/0886022X.2021.1906701

**Published:** 2021-05-10

**Authors:** Blaithin A. McMahon, Lakhmir S. Chawla

**Affiliations:** a Medical University of South Carolina, Department of Medicine, Medical University of South Carolina, Charleston, SC, USA; b Department of Medicine, Veterans Affairs Medical Center, San Diego, CA, USA

**Keywords:** Furosemide, acute kidney injury, stress test, renal replacement therapy, risk assessment

## Abstract

Loop diuretics are among the most widely used drugs worldwide and are commonly employed in the management of complications associated with acute kidney injury (AKI), namely volume overload and electrolyte management. The use of loop diuretics in critically ill patients with AKI is paramount to preventing or treating pulmonary edema. The naturetic response to a loop diuretic is based on its unique renal pharmacology. Our review article summarizes the pharmacology of furosemide in the intact nephron and discusses how this response might be altered by the presence of AKI. We discuss the increasing body of literature on the latest clinical utility of furosemide namely, it’s challenge test, known as the furosemide stress test which has highlighted a new and novel role for furosemide over the past number of years. This test assists with the identification of AKI subjects at higher risk of AKI progression and the need for renal replacement therapy. The stress test can also predict cessation of continuous renal replacement therapy in patients with established AKI. On the basis of the evidence presented in this review, we propose future potential studies of furosemide in AKI.

## Introduction

Acute Kidney Injury (AKI) is a common and serious complication that is associated with several adverse outcomes including death, need for renal replacement therapy (RRT), increased length of hospital stay, chronic kidney disease and rising health care costs [1,2]. Even mild forms of kidney injury influence short and long term morbidity and mortality through progression of chronic kidney disease (CKD) and cardiovascular disease [3]. Specific treatments for AKI are currently lacking and supportive care is the mainstay of therapy. Prevention is therefore of the upmost importance, and relies on the identification of individuals at high risk for development of AKI early in the intensive care unit (ICU) admission. Successful risk stratification in AKI is essential to identify patients at risk for progression of AKI (either progression to higher AKI stage or need for RRT) not only for therapeutic trials to minimize enrollment of mild cases of AKI but also to prepare patients for the impeding need for RRT or avoidance of future nephrotoxins (such as calcineurin inhibitors or intravenous contrast). Risk prediction scores and models, functional and damage biomarkers, kinetic eGFR, real–time GFR and the Renal Angina Index have all been developed to assist clinicians in their decision-making process when managing patients with AKI [4]. Despite significant advancement in disease risk prediction and outcomes in other fields of medicine, most of these risk stratification methods in AKI are infrequently used in the current era to predict the severity of AKI or the optimal timing of RRT initiation. Yet since the discovery of the first loop diuretics, ethacrynic acid and later furosemide, these agents have been used since the 1950s by physicians worldwide [5], mostly in the ICU, to assess the kidneys response or lack of response to a furosemide challenge as a clinical assessment of tubular function [6]. In this context, furosemide is being used to establish a triage decision for AKI progression. The recent developments to standardize the use of furosemide in the assessment of patients with early AKI has provided a novel dynamic functional test of tubular function [6]. In this Review, we provide a brief overview of the pharmacology of loop diuretics and how this response might be altered by the presence of AKI. We review the recent literature on how loop diuretics can serve in the risk stratification assessment in early AKI. We discuss the role of the furosemide stress test in the risk prediction of AKI progression, examine its performance with AKI biomarkers, in the initiation and cessation of RRT. Finally, we give an overview of the future potential uses of this test in patients with AKI.

### Pharmacology of furosemide

**Figure 1.**
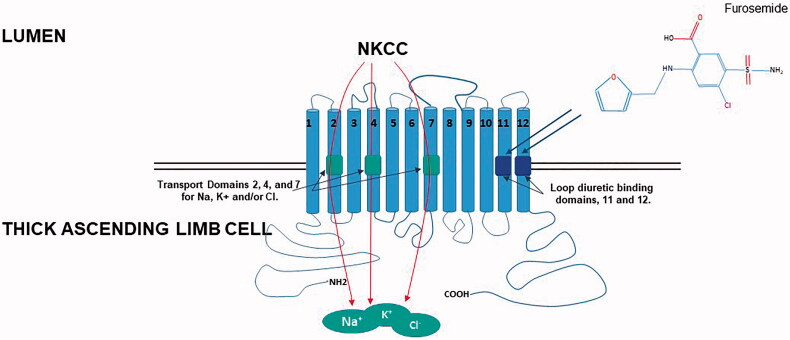
Schematic diagram showing the 12 transmembrane domain Na-K-2Cl (NKCC) transporter in the thick ascending limb of the loop of Henle. Loop diuretics bind to the chloride-binding site (portions of the transmembrane domains 11 and 12) resulting in obstruction and subsequent inhibition of the NKCC-2 transporter d*omains 2*, *4*, and *7* transport Na, K, and/or Cl.

Loop diuretics are among the most commonly prescribed drugs, ranking overall 15th most prescribed drug in the United States in 2014 [7]. Furosemide (4-chloro-*N*-(2-furyl-methyl)-5-sulfamoyl-anthranilic acid) is a potent natriuretic drug that is chemically synthesized and does not occur naturally (Figure 1). Furosemide exerts its actions by inhibiting the chloride binding domain located in the transmembrane domains 11 and 12 of the Na-K-2Cl cotransporter (NKCC-2) expressed in the thick ascending limb of the loop of Henle including the macula densa thereby inhibiting sodium reabsorption [8,9]. Inhibition of sodium reabsorption creates a concentration gradient in the renal medulla, which is the primary driver for more distal water reabsorption. In order for an abrupt natruriesis to occur in response to furosemide, a considerable amount has to occur prior to furosemide binding to the NKCC-2 cotransporter. Furosemide is absorbed quickly after oral administration with peak concentrations within 0.5–2 h, in contrast to intravenous administration which is within minutes. The duration of natriuretic effect is theoretically 6 h after oral administration (*la*sts *Six* hours) and 2 h after single-dose intravenous administration however these times can vary dramatically [10]. Absorption is slowed by food and edematous such as nephrotic syndrome or heart failure [11]. Loop diuretics are organic anions that are negatively charged and poorly lipid soluble allowing them to be highly bound to serum albumin (> 95%), therefore, limiting their filtration at the glomerulus and reducing bioavailability (approximately 50% for furosemide) [12].

To gain access to the peritubular region, loop diuretics must be secreted across the proximal tubule *via* binding to the organic anion transporters OAT1 and OAT3 on the basolateral membrane of the thick ascending limb cells. These organic acid secretory sites have an avid affinity for loop diuretics and free the diuretic from albumin. OAT 2 is also expressed on basolateral membrane but has a lower affinity for loop diuretics [13]. States of extreme hypoalbuminemia can interfere with the delivery of diuretic to the proximal tubule and can affect drug efficacy [14]. On the luminal side, multidrug resistance-associated protein 4 (Mrp-4) appears to mediate at least a portion of secretion into the tubular fluid. Knockout mice for OAT1, OAT3, and Mrp-4 are resistant to loop diuretics [15]. Uremic toxins, metabolic acidosis, hypokalemia, NSAIDs and cephalosporin’s all interfere with OAT1 and OAT3-mediated transport of loop diuretics to their site of action [13,16,17].

Once a loop diuretic is secreted into the tubular fluid it then reaches its site of activity at the NKCC-2 at the thick ascending limb of the loop of Henle. The tubular concentration of furosemide determines its natriuretic effect, and the urine concentration of furosemide has been used as a surrogate for the tubular concentration [10]. Once at the NKCC-2 site, furosemide binds within the translocation ion pocket embedded in the chloride-binding site (portions of the transmembrane domains 11 and 12) resulting in obstruction and subsequent inhibition of the NKCC-2 transporter (Figure 1). Expression of Na-K-2Cl is also evident in cytoplasmic vesicles, suggesting a reservoir of transporters for insertion into the membrane [14], a process that can be modified by vasopressin and PGE2 [18,19]. At least half of an administered furosemide dose is excreted unchanged into the urine, a process that is prolonged in kidney failure (half-life of furosemide increases) [5] and the other half undergoes renal conjugation to glucuronic acid.

### Pharmacology of furosemide in critically ill patients with acute kidney injury

The furosemide clinical pharmacokinetic/pharmacodynamics (PK/PD) profile in critically ill patients with AKI is poorly understood and much of the data is extrapolated from patients with chronic kidney disease [20–22]. The bioactions of furosemide differs considerably in patients with AKI, especially in patients with advanced AKI and oliguria and is summarized in Table 1. Several factors account for these differences. Firstly, critically ill patients are often moderately to severely hypoalbuminemic. Furosemide protein-bound fraction is significantly reduced in hypoalbuminemic states and consequently reduces tubular secretion and the clinical efficacy of furosemide. Administration of IV albumin with furosemide can increase urine output up to six hours after albumin administration but it’s use for this purpose is controversial as its effects are short-lived and can be expensive [23,24]. Secondly, acute tubular injury often results in loss of epithelial polarity with redistribution of Na/K + ATPase from the basolateral membrane to the apical membrane [25]. This impairs sodium gradient across the tubule impairing secondary active transport of organic acids including the urinary excretion of furosemide impairing the urinary response to furosemide creating furosemide resistance. Thirdly, furosemide pharmacodynamics is changed in oliguric AKI patients, limiting urinary excretion of furosemide and therefore contributing to potentially harmful plasma levels of furosemide [26]. Fourthly, uremic organic acids and moderate to severe metabolic acidosis may theoretically compete with furosemide at the OAT1/OAT3 transporter site and thereby limiting furosemide secretion and activity [27,28]. Fifthly, OAT1/OAT3 transporters are dramatically increased in the urine of patients with AKI, and those with severe AKI may have additional OAT4 excretion in the urine. The presence of OAT4 transporter in the urine of AKI patients may indicate the severity of tubular damage and destruction and impair furosemide secretion into the urinary space [29].

**Table 1.** Altered bioactions of furosemide in critically ill patients with acute kidney injury.

Target abnormality	Mechanism	References
Hypoalbuminemia	Impaired furosemide delivery to nephron	23,24
Loss of epithelial cell polarity	Redistribution of Na/K + ATPase, impairing secondary active transport of organic acid.	25
Oliguria	Toxic plasma levels of furosemide	26
Uremic organic acids accumulation	Competiton with furosemide at the OAT1/OAT3 transporter site	27,28
Severe metabolic acidosis	Competiton with furosemide at the OAT1/OAT3 transporter site	27,28
Elevated OAT1/OAT3 (+/−OAT4) transporter secretion in urine	Tubular damage and destruction	29
Vasopressin administration	Increase expression of NKCC2 transporter and promote insertion into TAL of loop of Henle	18,30
Cephalosporin administration	Competition with furosemide at the OAT1/OAT3 transporter site	27,28

Finally, critically ill patients with AKI may receive exogenous source of vasopressin or its analog which can increase expression of NKCC2 transporter and promote insertion of this transporter *via* a cAMP-dependent mechanism at the thick ascending limb of the loop of Henle and macula densa [18,30]. The net effect is increased sodium and water retention. These mechanisms might have the effect of amplifying the defect in water excretion in edematous AKI patients.

Based on the pharmacological properties above, furosemide is an ideal functional tool to assess renal tubular integrity in AKI and has also been used in the assessment of early initiation of dialysis, prediction of delayed graft function in patients undergoing deceased-donor kidney transplantation and renal recovery as outlined in Table 2.

**Table 2.** Summary of furosemide challenge tests.

Study population	Study type	N,	Definition of FST used	Clinical Outcome	Ref
Adult patients with early AKI (stage I)	Prospective	54 92	IV furosemide 1 mg/kg in diuretic naïve patients and 1.5 mg/kg in non-naïve patients.	UOP <200 ml 2 h post furosemide predicts severe stage 3 AKI	6,31
Patients without AKI, FWC between +15 and −15 ml/h, falling UOP, fluid excess	Prospective	21	IV furosemide 80–160mg	UOP >200/h, Urine Osm from 600 mOsm/l–400 mOsm/l, no change in FWC and subsequent development of AKI	32
Pediatric cardiac surgery patients without AKI	Retrospective	568	Postoperative IV furosemide (0.8–1.2 mg/kg per dose between 8 and 24 h after cardiac surgery	Lack of furosemide responsiveness defined a priori as UOP < 1 mL/kg/h after furosemide predicted subsequent development of AKI	33
Pediatric cardiac surgery patients without AKI	Retrospective	166	IV furosemide (median dose of 0.9 mg/kg) administered 14 hours post cardiac surgery	The 2- and 6-hour urine flow rates were significantly lower in patients in whom AKI developed	34
Pediatric cardiac surgery patients without AKI	Retrospective	99	Postoperative IV furosemide (mean dose 1.1 +/− 0.3 mg/kg) with median of 7.7 hours after cardiac surgery	Lack of furosemide responsiveness defined a priori as 6-h UOP less than or equal to 5.6mL/kg predicted fluid overload and prolonged peritoneal dialysis	35
Non AKI and early AKI patients	Subanalysis of Prospective Observational study	95	Variable furosemide dose used. Furosemide responsiveness (FR) was defined as total UOP in 2 h (mL) divided by the dose of bolus furosemide (mg) administered.	FR could predict AKI progression in patients with high plasma NGAL levels (>142 ng/mL), while few patients with low plasma NGAL levels exhibited AKI progression	36
All stages of AKI	Prospective multicenter pilot study	162	Intravenous furosemide (1 mg/kg in furosemide-naive patients or 1.5 mg/kg in previous furosemide users). FST-nonresponsive patients (urine output less than 200 mL in 2 h) were randomized to early (initiation within 6 h) or standard (initiation by urgent indication) RRT.	AKI: timing of RRT initiation. Only 6/44 (13.6%) FST-responsive patients ultimately received RRT while 47/60 (78.3%) nonresponders randomized to standard RRT either received RRT or died (*p* < 0.001).	37
Patients with severe AKI on CVVH	Prospective single center randomized placebo-controlled study	71	After cessation of CVVH the first 4 h of urine was collected for measuring creatinine clearance. Patients were subsequently randomized to furosemide (0.5 mg/kg/h) or placebo by continuous infusion.	Furosemide by continuous infusion in the recovery phase of hemofiltration-dependent AKI did increase urinary volume and sodium excretion but did not lead to a shorter duration of renal failure or more frequent renal recovery.	38
Patients undergoing DDKT	Retrospective	200	Furosemide (one time intraoperative dose of 100mg) can predict delayed graft function post deceased donor kidney transplantation (DDKT). Furosemide can predict delayed graft function post DDKT transplantation	The FST predicted DGF with an area-under-the curve of 0.85 at an optimal urinary output cutoff of <600 mL at 6 h.	39
Patients undergoing DDKT	Prospective	59	Single dose of intravenous furosemide, 1.5 mg/kg at 3 h after allograft reperfusion. Urine volume recorded hourly after FST until 6 h. FST was compared to urine NGAL.	The 4-h urine volume less than 350 mL (FST non-responsive) was the best cutoff value in predicting DGF with 87.5% sensitivity, 82.9% specificity, and 82.5% accuracy. The FST is a more accurate biomarker than urine NGAL	40

FST: furosemide Stress test; KT: kidney transplantion; DDKT: deceased donor kidney transplantation; AKI: acute kidney inury; RRT: renal replacement therapy; DGF: delayed graft function; UOP: urinary output; FWC: free water clearance.

#### Testing of renal tubular integrity with furosemide in early AKI: Standardization of the furosemide stress test

**Figure 2.**
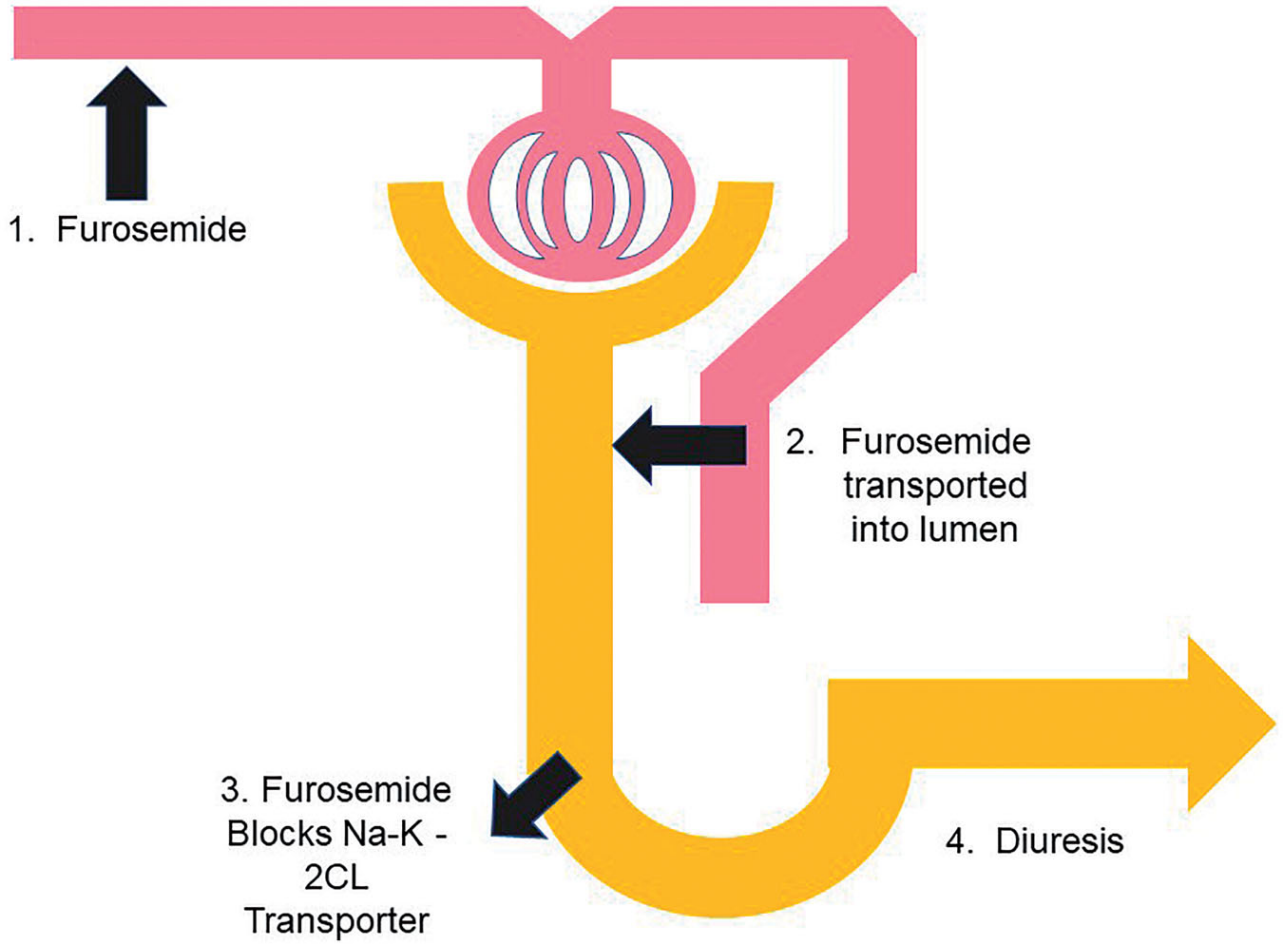
Testing of renal tubular integrity with the furosemide stress test in early AKI. In order for a brisk urinary response to furosemide there are four components that must be achieved. 1. Furosemide enters the blood stream and then must bind to albumin. 2. Active secretion by proximal tubular from the basolateral membrane to the lumen by the hOAT system. 3.Transport of the furosemide in the lumen dissolved in the glomerular filtrate transported to the TAL and binding to the Na- K-2Cl apical transporter. 4. Resultant diuresis.

Furosemide is likely more ideal than other loop diuretics (such as torsemide and bumetanide) to predict renal tubular integrity given that furosemide is not filtered and thus its function is completely dependent on tubular function (Figure 2). In this context, furosemide-induced urine output could be used a marker of AKI severity. This methodology to assess the integrity of the renal tubular function in the setting of AKI was initially described in 1973 by Baek et al. in 21 patients who received non standardized dose of intravenous furosemide [32]. 15 of 21 patients had poor response to furosemide as assessed by lack of change in urinary output, urine osmolality and free water clearance and subsequently went on to develop acute renal failure. This approach was then standardized in 2013 and has since been coined the *furosemide stress test* (FST) [6]. In this study, the FST was studied in a 77-subject pilot cohort (a mix of retrospective (*n* = 22) and prospective (*n* = 54) patients) whereby critically ill patients with early stage 1 or 2 Acute Kidney Injury (AKIN stage 1 defined as 6 h of oliguria (<0.5 mL/kg/h) or 0.3 mg/dL increase in serum creatinine or increase of 150–200% above baseline serum creatinine, or AKIN stage II (12 h of oliguria (<0.5 mL/kg/h) or increase of 200–300% above baseline serum creatinine) to predict progression to AKIN stage III (need for RRT or increased sCr to three times baseline or urine output less than 0.3 mL/kg/h.). Patients were challenged with a one-time dose of intravenous furosemide (1.0 mg/kg for loop diuretic naïve patients and 1.5 mg/kg for those who had prior loop diuretic exposure). This pilot study demonstrated that the 2-h urine output in response to a furosemide challenge was able to predict progression to Stage 3 AKI with in-hospital mortality and a composite of death or progression serving as secondary end points. A urinary response of less than 200 mL over the first 2 h post furosemide administration provided an area under the receiver operating curve (AUC) (standard error [SE]) of 0.87 (0.05) with a sensitivity and specificity of 87% and 84%, respectively, for the progression to AKIN Stage 3. More importantly, it was determined that the FST was feasible, safe and well tolerated in critically ill patients [6].

Since publication of this initial pilot study, there have been several retrospective validations of this cutoff (described below) and more recently the publication of the multicenter prospective study [31]. The multicenter study recruited patients from ICUs at 5 academic centers in the United States and Canada from January 2014 to August 2017 enrolling 92 patients. There was a significant higher portion of patients with stage I AKIN in the prospective study compared to the pilot study. There were a higher APACHE II scores in the progressors vs. non-progressors (22.1 [1.71] vs. 18.9 [1.03], *p* = 0.10), lower baseline eGFR (56.8 [4.79] vs. 67.9 [3.66], *p* = 0.14) and higher baseline sCr (1.27 [0.08] vs. 1.22 [0.06], *p* = 0.34), albeit none achieved statistical significance. The multicenter study found similar operating characteristics for the FST with a urinary cutoff of 200 mL over the first 2 h (sensitivity 73.9% and specificity 89.9%) [31]. Enrollment was challenging and the incidence of hypotension was 9.8%, almost double that in the pilot study suggesting that the FST should not be utilized in hypovolemic patients. There were no critical life-threatening events recorded in the multicenter study.

#### Furosemide responsiveness predicts AKI in children after cardiac surgery

Over the past 2 years, three retrospective studies have been published validating the furosemide responsiveness in cohorts of pediatric patients after cardiac surgery [33–35]. In one study by Penk et al. retrospective assessment of 166 pediatric patients from 4 medical institutions (median age, 6.3 months) were included with an AKI rate of 54/166 (33%) [34]. Urine output was recorded hourly after the first dose of furosemide after surgery. Lower urine flow rate after furosemide administration was independently associated with subsequent AKI. 2- and 6-h urine flow rates were significantly lower in patients who developed AKI: 2.9 (0.9–6.5) versus 5.0 (2.5–9.0) mL/kg/h for 2-h urine flow rate, *p* = 0.004, and 2.4 (1.2–4.0) versus 4.0 (2.3–5.9) mL/kg/h for 6-h flow rate, *p* = 0.001. Future prospective studies to validate the FST as a predictor of AKI post cardiac surgery is warranted.

#### Performance of FST compared with AKI biomarkers

The performance of the FST has been compared with kidney damage biomarkers to predict AKI progression. In the study by Matsuura et al. retrospective analysis of 95 patients with AKIN stage I and II, compared the FST with the prognostic characteristics of plasma NGAL to predict severe stage AKIN III. Furosemide responsiveness was determined to be the better predictor of progression to AKIN stage III (AUC 0.87, 95% CI 0.73–0.94) and had better operating characteristics compared to plasma NGAL (AUC 0.80, 95% CI 0.67–0.88) [36]. In the initial FST pilot study by Chawla *et al.*, 2013, the 2-h urine output after FST was significantly better than each urinary biomarker tested (IGFBP7/TIMP2 and urinary NGAL) in predicting progression to Stage 3 AKI (*p* < 0.05) [41]. In addition, the performance of the FST was enhanced when utilized in patients with increased levels of AKI biomarkers.

#### FST and the timing of initiation of renal replacement therapy

The FST could be used to screen patients at high risk for RRT and has been used in decision making to guide the timing of initiation of RRT, in particular in clinical trials to enhance enrollment of RRT timing. Lumlertgul et al. [37] conducted a prospective, multicenter, open label trial using the FST in 162 patients with all stages of AKI in an effort to exclude low-risk patients from enrollment into trials of RRT timing. In order to randomize patients to either early or standard RRT initiation, 44 FST-responders (patients who made more than 200 mL in the first 2 h) were excluded from randomization, while 118 were FST non-responsive and randomized to either early or standard RRT initiation. Only 6 of 44 (13.6%) FST-responders subsequently underwent RRT. Furthermore, FST-non responders were highly predictive of requiring RRT with 45 of 60 patients randomized to standard RRT initiation, receiving RRT. In this manner, the FST was used a risk-stratification method to tease out the severe cases of AKI among all cases of AKI that have a higher need for RRT and avoided enrollment of low risk patients into a clinical trial of timing of RRT.

#### FST and the prediction of renal recovery

The furosemide response has been used to predict renal recovery and cessation of RRT in critically ill patients recovering from AKI [38]. In one study, urinary output was measured over a 4-h period after cessation of CRRT in 71 critically ill patients and was measured again 24 h later following either continuous intravenous furosemide 0.5 mg/kg/h or placebo administration [38]. Eighteen of the 71 included patients showed immediate recovery of renal function over the initial 4-h episode, defined as measured urine creatinine clearance >30 mL/minute and did not require further CRRT. 25 patients received furosemide and 24 h later a 4-h urine portion was again collected. This second portion also showed a significantly higher urine production (654 (333–1155) ml) in those patients in whom renal recovery occurred eventually during their hospital stay compared with those who did not recover (48 (15–207) ml, *p* = 0.007), resulting in an AUROC of 0.84.The 36 furosemide-treated patients had a significantly increased urinary volume compared with the 35 placebo-treated patients (median 247 mL/hr. (interquartile range [IQR] 774 mL/h.) vs. 117 mL/h. (IQR 158 mL/h.), *p* = 0.003). In 25 patients in the furosemide group and 27 patients in the placebo group showed recovery of renal function at ICU discharge (*p* = 0.46). This recovery work along with data from heart failure patients (independent of AKI status) points to the utility of the response to furosemide as a clinically useful prognostic biomarker for adverse patient outcomes. The performance of the FST in adults to predict both severity of AKI and the recovery on AKI is remarkably consistent and the ROC AUCs are typically greater than 0.85 (Table 2). As such, this safe inexpensive functional test performs across all AKI stages and the time to the study result is as short as 2 h.

#### FST in predicting delayed graft function in renal transplant recipients

The FST has been used in the prediction of delayed graft function (DGF) post deceased donor kidney transplantation (DDKT) [39]. In this retrospective study of 200 patients, the urinary response to a single intraoperative dose of 100 mg of intravenous furosemide after the anastomosis of the renal vessels predicted the need for RRT at 2 and 6 h post kidney transplantation (KT). Patients with DGF had a significantly decreased urinary response to intraoperative furosemide (73 mL vs. 250 mL in 2 h following the FST, *p* < 0.001). Additionally, the 6 h urine output of 500 mL after the FST provided an AUC of 0.86 providing a sensitivity and specificity of 85% and 76% [39]. In a small prospective study of 59 patients undergoing DDKT, the urinary output after administration of furosemide 1.5 mg/kg 3 h after allograft reperfusion was used to predict DGF (requirement of dialysis within one week of KT) and its response was compared to the prognostic characteristics of urine NGAL [40]. The 4-h urine volume less than 350 mL was the best cutoff value in predicting DGF with AUC of 0.939 (CI 0.882–0.996), 87.5% sensitivity, 82.9% specificity, and 82.5% accuracy. The FST outperformed urine NGAL for the prediction of DGF AUC 0.733 (0.586–0.880). These data suggest that the FST may be used as a clinical prediction tool for the future need of RRT, not only in general medical/surgical patients, but also in those patients undergoing kidney transplantation.

#### Deconstruction of the FST

The FST effectively interrogates the nephron with a focus on tubular function and a clinical readout which is the urine output over a period of time (typically 2 h). When the FST is deconstructed, it can be sub-divided into four components (Figure 2). To date, the FST has been tested as an integrated test: furosemide administered and then the urine output is assessed. However, natriuresis rather than urine output in response to loop diuretics may also be an equally important prognostic sign in AKI but has yet to be determined. In acute decompensated heart failure patients, it has been shown that natriuresis is more prognostic of six month mortality [42] than urinary output and this approach is now endorsed by the Cardiorenal Working group of the European Society of Cardioloy [43]. In addition, measurement of urine sodium and urine furosemide may add prognostic information. The additional measurement of urine furosemide, most easily done as assessing the percentage of the furosemide excreted mass (FEM) – [the amount of furosemide secreted divided by the mass of furosemide administered multiplied times 100], would inform the clinician as to the proximal tubular secretion capacity. In most patients, one would expect the urine output and FEM to be strongly correlated, but in the circumstances where this not the case, this may suggest proximal versus distal injury, or vice versa depending on the nature of the discordance. Similarly, knowledge of the urine sodium, particularly when the FST results in poor diuresis would be potentially informative. If the urinary sodium concentration was low and the FEM was high, this would suggest that there is enough renal blood flow to maintain intact bi-directional tubular function, but would suggest inadequate circulating volume as commonly seen in volume depletion, heart failure, and cardiorenal syndrome with increased central venous pressure. Whereas, elevated urine sodium and decreased FEM would be more suggestive of tubular injury (Table 3).

**Table 3.** Proposed utilization of urinary furosemide and urinary sodium with decreased urine response to FST.

	Urinary furosemide (FEM_2_)	
	FEM high	FEM low
Urine Sodium		
High Sodium	Physiology: Consistent preserved proximal tubular function and distal injury preventing sodium reabsorption Syndrome: This profile would be consistent with kidney injury that has a TAL or distal injury profile	Physiology: Consistent with tubular injury resulting inability to reabsorb sodium or to secrete furosemide Syndrome: This profile would be consistent with acute tubular injury and acute tubular necrosis
Low Sodium	Physiology: Demonstrates sufficient oxygen transport to the kidney to enable effective bi-directional proximal tubular function as evidenced by sodium reabsorption and furosemide secretion Syndromes that would show this pathophysiology: decreased effective circulating volume, congestive heart failure, cardiorenal syndrome, central venous congestion	Physiology: Demonstrates sufficient oxygen transport to the kidney to maintain sodium reabsorption but inability to secrete furosemide. Syndrome: This physiology would be consistent with a profound hypo-albuminemia state and may be indicative of the threshold wherein albumin replacement therapy is indicated

The use of urinary sodium concentration and FEM to enhance the diagnostic capacity of the FST is theoretical and requires validation. The suggested use of urinary sodium and FEM is a proposed framework on how to expand the utility of this test.

## Future potential for FST in the assessment of chronic kidney disease

CKD is a serious public health hazard worldwide and risk assessment and prognostication is typically conducted by measuring equilibrated GFR (eGFR) and level of proteinuria. Both these tools have significant limitations because the eGFR equation requires standardized creatinine measurement and can have higher levels of bias at higher level of GFR. Proteinuria is an excellent predictor of risk, but many patients with CKD do not have significant proteinuria. In CKD, the development of kidney fibrosis is considered one of the most potent risk factors for renal disease progression, independent from the CKD etiology, as it represents a final common pathway from injury [44]. In aggregate, the tubules and interstitial compartment comprise over 75% of the renal mass and therefore an understanding of the degree of fibrosis is critical. The main limitation is that currently, kidney fibrosis can only be assessed *via* kidney biopsy which may be too invasive for routine serial assessment. However, we postulate that since tubules comprise the majority of renal mass and reduced kidney size correlates with worsening CKD, that tubular atrophy and fibrosis are a part of the fibrotic pathway and represent the loss of tubular function. Thus, measures of tubular function and reserve are likely to be robust measures of fibrosis in CKD.

Some evaluation tools to assess tubular function have been tested such as dilution capacity, concentration [18–30,32] and urinary acidification [31,32,34] ,in addition to excreted fraction of sodium and urea (FENA and FEBUN) [33,35,36], however these have not been widely used in clinical practice.

We postulate that the FST may be a useful tool to evaluate tubular function and reserve and may be able to serve as a noninvasive tool to assess kidney fibrosis. We have conceived the concept of the furosemide excreted mass at 2 h (FEM_2_). Similar to the forced exhaled volume (FEV_1_) in pulmonary function testing, a healthy person can expire most of the air from their lung in 1 s. Whereas a patient with lung disease often demonstrates a decreased FEV_1_. Similarly, a healthy person with a full complement of functioning nephrons should be able to promptly eliminate furosemide into the urine. However, when tubular dysfunction is present as the case with tubular-interstitial fibrosis, the fibrosed kidney will not be able to efficiently move furosemide out of the plasma and into the tubular lumen. This type of functional testing may form the basis of assessing tubular function in a noninvasive fashion.

In summary, the we posit that the FEM_2_ would decrease in a step-wise fashion in patients with increasing fibrosis and CKD as compared to healthy patients with normal kidney function (Table 3). Further, we would expect fibrosis levels and FEM_2_ would correlate with eGFR but offer distinctive prognostic information. Further research is warranted to validate these concepts and may require correlation with patients who have undergone kidney biopsy.

### FST use in the assessment of critically ill COVID-19 patients with AKI

Caution on the use of the FST in critically ill COVID-19 patients with AKI should be emphasized here. There is a bimodal onset of AKI in patients with COVID-19 – those patients who are hospitalized on admission with AKI and those who acquire AKI during their hospitalization [45]. COVID-19 patients with AKI on admission regularly have hypovolemia due to high insensible losses from fever, acute viral prodromal phase of illness, GI disturbances and poor oral intake. It should therefore be advised that the FST be avoided in patients with volume depletion to ensure hypotension is evaded. Only after the fluid resuscitation phase of AKI is addressed should the FST be contemplated. This can occur in an isovolemic manner with matched urine output and hydration protocols.

### Further potential for the FST

Other aspects of the FST that merit further research include assessment with other loop diuretics other than furosemide (e.g., torsemide, bumetanide) especially given increasing use of these loop diuretics in the context of acute heart failure. In addition, further studies on the use of the FST in later stages of AKI other than early stage AKI is justified. Frequently, patients with severe AKI receive a high dose of intravenous furosemide to prevent or treat oliguria. This information is frequently used to predict if a patient is a ‘responder’ or ‘non-responder’ to furosemide and the subsequent need for RRT. This approach should be standardized in advanced stage 3 AKI to avoid delayed definitive therapy or RRT and may be clinically relevant use of the FST when compared to it’s use in early stages of AKI. In conclusion, the FST has been demonstrated to safe and effective predicting the severity of AKI across multiple cohorts and investigators over three continents and its overall performance has been consistent. In addition, the FST appears to have the capacity to predict severity in sub-types of AKI such as DGF and also appears to predict recovery. FST may also have a role as a diagnostic in CKD.
